# Atlanta Academy of Medicine

**Published:** 1872-08

**Authors:** J. T. Johnson


### ATLANTA ACADEMY OF MEDICINE.

J. T. JOHNSON, M.D., REPORTER.

[Extract from Minutes, January 22d, 1872—Dr. J. P. LOGAN in the Chair.]

Dr. W. H. Cumming referred to a case treated by a man of this city, who makes pretensions as an oculist. The patient is a boy, aged thirteen, who had his eye penetrated, five years ago, by a fragment of a percussion cap, which cut the cornea and iris one or one and a half lines from the sclerotic; since that time there has been constant trouble and irritation. The probability is, that the fragment still remains in the eye. This man has expected to cure the eye by the application of his one remedy, nitrate of silver. The question of interest is, will the left eye, now sound, remain so? Since vision of the injured eye is destroyed, should it not have been enucleated. This would have prevented sympathetic ophthalmia, which may yet supervene.

Dr. W. F. Westmoreland has seen, during the past year, some reports of a cure of the ophthalmia by enucleation of the diseased eye. Would like to hear from Dr. Simpson, as he has just returned from the special study of the diseases of the eye, in New-York.

Dr. C. A. Simpson stated that it was his belief, that if the ophthalmia was recent, it could be cured by the enucleation.

Thinks it safer to remove the injured eye when vision is destroyed, or as least so soon as the sound eye is threatened. In the hospitals, this danger was always watched for closely, and if discovered, enucleation performed. Has seen in two instances, by the aid of the ophthalmoscope, a piece of metal lodged in the back part of the eye, and yet there was neither irritation nor sympathetic disturbance.

Dr. W. F. Westmoreland.—If the disease in the opposite eye has not become incurable, should operate; otherwise not, unless to relieve pain.

Dr. W. H. Cumming.—The best authorities in Europe think it almost an invariable rule, that the eye can not be cured after the disease has so far advanced as to be recognized. Hence, the eye should be removed at the time of injury, if destroyed, as sympathetic disturbance of the other can be always thus prevented. In the case discussed, the eye should be now evacuated, as the left one is still good.

Dr. C. A. Simpson.—It is good practice to enucleate in this instance, as irritation is always present in the injured eye. Surgeons frequently wait till the sound one becomes involved, if there should be any hope of saving the injured one.

Dr. W. H. Cumming.—Had had fine opportunity, while in China, of witnessing the destructive effect of this sympathetic inflammation. Must have seen as many as two or three hundred cases of total blindness so produced.

Dr. W. A. Love.—Does the diseased eye discharge any pus? Thinks that copper will continue to produce such a discharge, rather than become encysted. When a boy, had a piece of percussion cap driven into his finger; it remained imbedded in the periosteum for many years, till he became a medical student. During all this time, it was a source of irritation, with occasional accumulations of pus.

Dr. W. F. Westmoreland detailed a case of a man, aged near sixty, who had been for a long time passing calculi the size of a grain of wheat, or even as large as a pea. There was a stricture of the urethra, resulting from perineal section for abscess, three years before. Some months ago, one of these calculi lodged in the membranous portion of the urethra, in the pouch behind the stricture. He attempted to dilate the stricture, but abandoned the design in a week or two, the pain caused by the calculus preventing its success. He then tried the little hooks, but they always slipped back into the pocket. He then passed Sir Henry Thompson's dilator. The blades were opened by turning the screw, the calculus slipped between them, and was removed at the first attempt. Three were extracted in this way. Regarding it as something new, he wrote concerning it to Dr. Gouley, of New-York, who, in his reply, approved this use of the instrument, stating that he had not heard before of such an application of it. Sir Astley Cooper used an instrument something similar to this in construction. Dr. Gouley has improved the one here used by passing through the tunnel, in the curved end of it, his fine filiform, whalebone bougies; by the aid of these, the stricture often can be passed. As many of them as may be necessary can be introduced.

Dr. Baird stated that he had seen Dr. Gouley have as many as a half dozen of these bougies in the urethra at one time. When one became entangled in a sack or follicle, instead of wiihdrawing it, he would immediately introduce another. He would proceed in this way till the sacks were filled and the stricture passed.

Dr. Westmoreland exhibited the instrument, and the mode in which he used it.

Dr. Logan (Dr. J. G. Westmoreland in the chair) detailed a case recently treated by himself. The patient, an adult male, had been exposed a great part of the night in watching his premises for depredators. The symptoms were somewhat similar to those of meningitis, though not decided. During the latter part of the night, he was seized with a child. When he reached the patient, he was insensible—could not be aroused at all. Placed him on large doses of calomel and quinine. His condition rendered it difficult to administer the medicine, but in a few hours, he had taken as much as thirty-five grains of the first, and forty grains of the latter. Also used counter-irritants to the spine. The patient remained in this condition for forty-eight hours. There was no spasm or rigidity of the muscles. At the expiration of this time, after some copious evacuations, he emerged from this condition of coma. Rheumatism was now developed in a number of the joints, with swelling, redness, etc. Patient is now nearly well of the rheumatism. Has not met with any case like it. Was in doubt as to whether it was cerebro-spinal meningitis, with a manifestation in the joints, or rheumatism first affecting the brain. About the third day, the patient proved to be ptyalized.

Dr. Wells.—What was the condition of the skin?

Dr. Logan.—During the time while the patient was in the condition of stupor, the heat seemed to be less than natural. The pulse was not decidedly weak, but seemed to lack tone; it afterward became strong.

Dr. John Stainback Wilson.—Was there anything peculiar about the tongue?

Dr. Logan.—Nothing special.

Dr. Cumming.—This seems to be a case where the patient was overwhelmed by some poison in the blood. Two years ago, saw a case where the man seemed utterly crushed at once by the poison of syphilis. He was affected with albuminuria; the discharge of urine was very variable, and with this varied the condition of the patient. His vision was also affected. One day, found him insensible for twelve or fifteen hours. Thinking it was from inaction of the kidneys, used hot applications, dry cups, etc. He emerged from this stage, but some time afterward was again similarly attacked, and again improved, with discharge from kidneys as before. Acetate of potassa was also administered on these occasions. He afterward had a third attack, and died, the physician in attendance not knowing his previous history.

Dr. Rauschenberg.—In an article that I recently translated, out of one hundred and three cases of meniningitis observed in Greece, there was in every case a distinct swelling of some of the joints. There seems to be a something in the blood, and this to be an effort at eliminination.

Dr. Logan.—Some of the members have been for a long while of the opinion that epidemic meningitis is of the same nature with influenza, and that the poisonous influence may have various developments—as, for example, tonsillitis, pleuritis, etc.; that this influence, or this poison, does not always affect the brain or the spine, but that in a large proportion of the cases affected by the influenza, we have some other more common development. Has himself become so convinced, from his observation of the epidemic.

Dr. W. F. Westmoreland.—Thinks that this case of Dr. Logan's was one of meningitis. Similar to the symptom alluded to by Dr. Rauschenberg, was the frequent occurrence, in the epidemic last winter, of pleurodynia as a premonitory symptom. Has been long convinced that meningitis and influenza are of the same nature. Doubts if there be inflammation of the spine in many of the cases—that is, in those dying in a very few hours from the time of attack. In such cases, death occurs without any organic lesion of the brain. If the patient should live long enough, then this influence, or poison, may produce that inflammation. Has doubt if the disease is not misnamed.

Dr. Wells.—But do we not find effusions at the *post-mortem* in all instances?

Dr. Westmoreland.—I allude to those cases where the patient is stricken down in a few hours. There is not time for the inflammation to have become developed. I once saw a medical student stricken down in a few hours by the cholera poison. He was at the time in the wards of a hospital, and died before he could be removed, having but one evacuation from the bowels. It was not the discharges that killed him, but the intensity of the cholera poison. So, in meningitis, often it is not the inflammation of the membranes that kills the patient, but the poison itself that overwhelms him.

Dr. Cumming.—When a man dies in twelve hours, we find changes in the brain to account for the convulsions that usually have occurred; and we find evidence, too, that the inflammation has existed for probably seventy-two hours. But the man has been unconscious of that inflammation, or of any change; so that the mau is really killed in apparent health—is virtually a dead man when the first symptoms are manifested; he is a corpse in twelve hours, or even less, from the time of the first headache.

Dr. W. F. Westmoreland.—I deny that these lesions can exist without symptoms.

Dr. Wells.—I would ask this question, Is it possible for death to occur without the effusion?

Dr. W. F. Westmoreland.—There is no lesion in these cases where death takes place in so short a time. We will find them if the patient live long enough after attacked; and the longer he lives, the greater are the changes.

Dr. Cumming.—This does not accord with the observation of physicians, as we find it recorded in books. The disease has been studied closely in many localities, especially so in New-York. In the instances where the cases lasted only twelve hours, there were found such lesions as required seventy-two hours for development.

Dr. Bozeman then adduced examples from cholera, scarlatina, etc., to prove how death may occur from an overcharge of the poison before there has been time for the development of pathognomonic lesions.

Dr. W. A. Love referred to his study of the disease before the war, as reported in Dr. Hay's journal, in which pus cells were claimed to have been found after an illness of only a few hours. It was afterward proven that these were not really pus cells, but degenerated blood corpuscles.

Dr. Baird inquired of Dr. Logan, if the urine was examined in his case? It appeared to him that it may have been uræmia. The chill and insensibility were present, but there was no opisthotonos, or muscular rigidity, to indicate meningitis. Was there any vomiting?

Dr. Logan.—There was nothing peculiar about the urine to call attention specially to it. There was no vomiting.

Dr. Wells.—Was there any dilatation of the pupils?

Dr. Logan.—There was limited dilatation, with loss of sight.

Dr. Wells.—Thinks, on the whole, it must have been meningitis. Perhaps his calomel and quinine cured him?

